# Surgical Description of Laparoscopic Ovum *Pick-Up* in Buffalo Calves

**DOI:** 10.3390/ani13010102

**Published:** 2022-12-27

**Authors:** Alysson J. de O. Sousa, Heytor J. Gurgel, Paula S. A. Coelho, Carla R. G. Silva, Luiz H. V. Araújo, Hamilton S. do Nascimento, Izamara do S. R. Rodrigues, Luciano C. Pantoja, Thiago da S. Cardoso, Maykon D. Silva, Ana Carolina C. Torres, Pedro Paulo M. Teixeira, Moysés dos S. Miranda

**Affiliations:** 1 Institute of Veterinary Medicine, Federal University of Pará (UFPA), Castanhal 66075-110, Brazil; 2 Federal Institute of Pará (IFPA), Castanhal 68740-970, Brazil

**Keywords:** IVEP, surgery, *Bubalus bubalis*

## Abstract

**Simple Summary:**

In modern livestock, using calves as embryo donors is already a reality; thus, animal genetics can be transmitted before these animals have reached sexual maturity, considerably reducing the interval between generations. This fact becomes more relevant in buffalo species, as they present great inconsistency in the results of in vitro embryo production. This study is the first to describe a safe and effective surgical procedure for obtaining oocytes by laparoscopy in a farm environment using three surgical ports and transabdominal needle aspiration.

**Abstract:**

The technique of laparoscopic oocyte aspiration has been increasingly used in animals; however, there are few records of its use in buffaloes. To describe this technique, six suckling Murrah buffaloes aged between 3 and 5 months were used. Three laparoscopic ovum *pick-ups* were performed in each animal, with intervals of 15 days between surgeries, completing a total of 18 procedures. The technique used three surgical ports with optics and a high-definition video camera. The introduction of the first portal and insufflation of the abdomen was performed through the open technique, with aspiration using a 20 G needle transabdominally and a vacuum pump calibrated at 50 mmHg. The mean complete surgical time from anesthesia to the removal of the animal from the litter was 49 ± 9.8 min. There were 27.8% cases of insufflation on the wrong side of the omentum. The oocyte recovery rate of 60.3% remained within the normal range. However, the rate of viable oocytes recovered was low, with only 40.8% of those recovered undergoing in vitro embryo production (IVEP). These data demonstrate that this simple, minimally invasive technique is an excellent reproductive tool for the genetic improvement of buffalo species.

## 1. Introduction

Laparoscopy has been used efficiently for a long time for oocyte and embryo collection, especially in small ruminants, particularly in sheep [1]. This technique for oocyte collection is called laparoscopic ovum *pick-up* (LOPU). The female’s ovaries are aspirated into the abdominal cavity, thus avoiding laparotomy and externalization of the uterus and ovaries, as well as maneuvers that cause significant postoperative complications due to excessive exposure of the abdominal cavity and specific organs to the external environment [2,3,4,5]. 

From a surgical viewpoint, small incisions (ports) typical for laparoscopy are sufficient to introduce atraumatic forceps (commonly 5 or 7 mm in diameter and 33 cm in length) and the laparoscope itself [3,4]. Some variations of the technique have been reported, such as the use of larger ports (5–10 mm in diameter) and a high-definition camera attached to the laparoscope, allowing visualization of the surgical procedure on a video monitor [5,6]. The LOPU technique is considered a simple procedure, without several complications and with a short learning curve, and it can be ideally used in the field. This technique has an average duration of 26.75 ± 9.6 min in sheep and is commonly used for this animal species [4,7].

In addition to sheep, LOPU has been used in the juvenile stages of large farm animals such as cattle and buffaloes since, in the adult stage, transvaginal ovarian follicular aspiration is well established and efficient in these species [4,8].

The first LOPU-IVEP procedures in buffalo calves were recorded in 2017 by Brazilian [9] and Canadian [10] researchers. The former reported the use of the technique in 10 buffalo heifers between 2 and 4 months of age, which, after LOPU, obtained an average of 10.88 ± 3.25 oocytes/animal, with 7.63 ± 2.69 viable oocytes/animal, generating five embryo transfer and two pregnancies at the end of the experiment [9]. The latter used eight Mediterranean buffalo heifers aged between 2 and 6 months. A total of 16.5 oocytes/animal were recovered, and 13.8 oocytes/animal were viable, resulting in 10 embryos that resulted in three pregnancies [10].

The laparoscopic ovum *pick-up* in buffalo heifers was recorded in later articles [11,12]. However, to date, no detailed description of this technique in buffaloes has been reported. Therefore, this study sought to describe LOPU in buffalo heifers by reporting the details of the surgical procedure and its complications, as well as by recording the average times of each step and its effectiveness through oocyte recovery.

## 2. Materials and Methods

### 2.1. Study Site and Animals

The study was conducted at Fazenda Galileia, located at coordinates 1°18′39.0″ S and 47°50′21.7″ W, city of Castanhal, Amazon region. Six buffalo heifers (n = 6) of the Murrah breed were randomly selected from a herd of 29 animals, aged between three and five months, with a mean body condition score of 2.5 on a scale ranging from 1 to 5, according to Abrahamsen [13], in which 1 is equivalent to a very thin animal and 5 is equivalent to an obese animal, with an average weight of 90 kg. The calves suckled in a calf-at-foot milking system, remaining for approximately 30 min after milking with their mothers, and then released on Mombasa grass (*Megathyrsus maximus*) pasture, where they remained until the next milking.

Each calf was submitted to LOPU three times, with an interval of 15 days between the aspirations, totaling 18 LOPU procedures.

### 2.2. Ethical Approval 

This study complies with Law 11,794 of 8 October 2008, with Decree 6899 of 15 July 2009, and with the rules issued by the National Council for the Control of Animal Experimentation of Brazil (CONCEA), and it was approved by the Ethics and Animal Experimentation of the Federal University of Pará (CEUA/UFPA 9492230520).

### 2.3. Surgical Assessment

Regarding the time record, the LOPU procedure was divided into four steps: (1) anesthetic and antisepsis procedure (AA), (2) establishment of laparoscopic ports and abdominal insufflation (PE), (3) ovarian manipulation and aspiration (OM), and (4) uterus and ovarian washing, deflation, and suturing (DS). The time of each step was recorded to determine the duration of each phase with the procedure’s total duration, and the LOPU learning curve was estimated.

### 2.4. Fasting, Anesthetic Procedure, and Antisepsis

**Figure 1:**
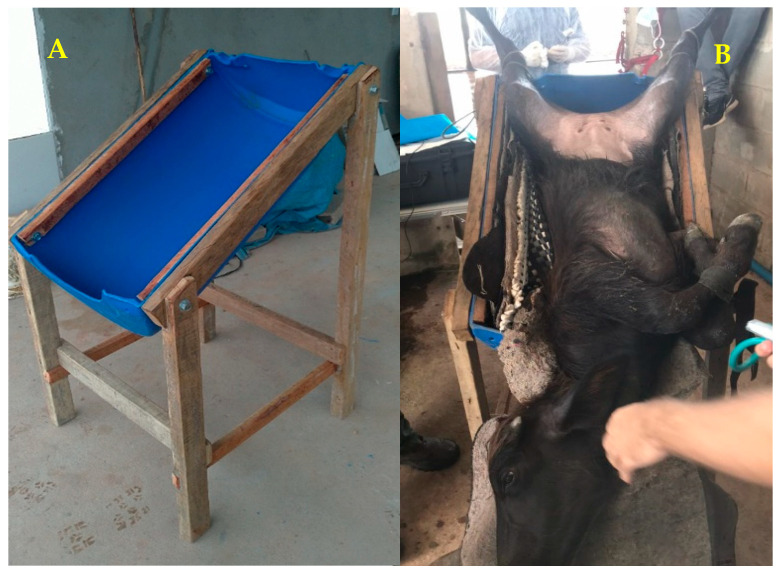
Stretcher adapted to perform the LOPU (**A**). Animal in Trendelenburg position at 45° (**B**).

**Figure 2:**
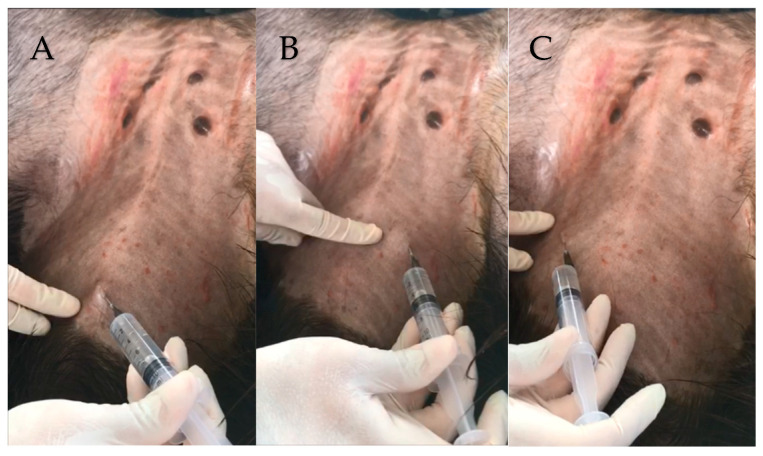
Performing local anesthesia, in the places where the first (**A**), second (**B**), and third (**C**) laparoscopic ports were to be established.

The animals were subjected to food and water fasting for 36 h and 24 h respectively. For performing LOPU, the animals were sedated with 0.2 mg/kg of 2% xylazine hydrochloride (Xilazin^®^, Syntec, São Paulo, Brazil), IM. After reaching the lateral decubitus position, the abdominal surgical area hair was clipped and the site was washed with soap and water, and then the animals were placed in a 45° Trendelenburg position on an adapted immobile stretcher (Figure 1). Routine skin antisepsis was then performed with 2% chlorhexidine gluconate (RIOHEX Clorexidina 2%^®^, Rioquimica, São Paulo, Brazil) and 70% ethyl alcohol. The infiltrative local anesthetic blockade was performed using 3 mL of the hydrochloride of lidocaine 2% (Lidovet^®^, Bravet, Rio de Janeiro, Brazil) at the sites where the trocars were introduced (Figure 2).

Anesthetic time was calculated from the xylazine application at the beginning of the incision. In addition, respiratory (RR) and heart (HR) rates were measured every 10 min from the time of sedative application until the end of the surgical procedure.

### 2.5. Establishment of Laparoscopic Ports and Abdominal Insufflation

The technique described here was based on previous studies [7], which proposed using three laparoscopic ports, two of which were 10 mm and one of which was 5 mm. It is highly recommended to use 5 mm trocars for the establishment of all laparoscopic portals; however, in this study, we used 10 mm trocars due to availability.

**Figure 3:**
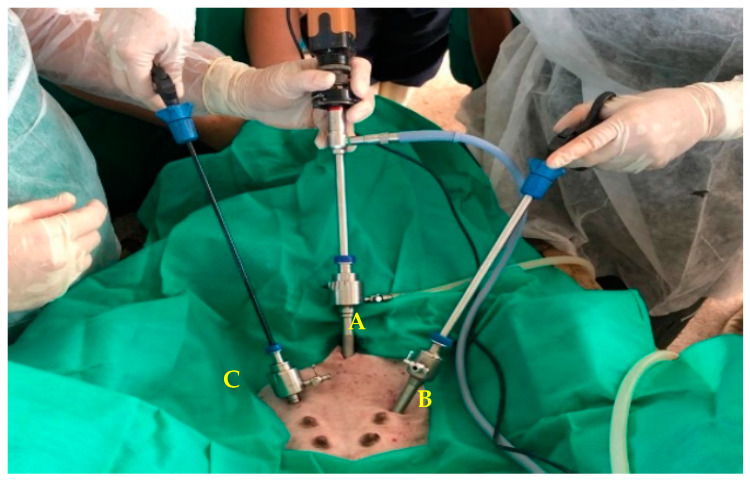
Arrangement of the laparoscopic ports in the caudocranial direction, with the animal in the supine position: first port with inflation valve and rigid optics (A), second port with 10 mm forceps (B) and third port with 5 mm forceps (C).

The surgical procedure began with an incision, with a scalpel, in the midline, approximately 10 cm caudal to the umbilical scar, followed by the introduction of the first trocar with a diameter of 10 mm (Ø) (Bhio supply^®^, Sapucaia do Sul, Brazil), using an open technique (Hasson). After removing the trocar obturator, an electronic insufflation device (Dyonics Access 40^®^, Smith and Nephew, Andover, USA) was attached to the trocar insufflation valve through a silicone hose. The abdomen was then inflated with CO_2_ under an intra-abdominal pressure of 10 mmHg and an inflation rate of 10 L/min. Once pneumoperitoneum was established, a 10 mm (Ø), 0° laparoscope was introduced and connected to a fiber-optic cable (Fiber optic 495 light cable^®^, Karl Storz SE & Co., Tuttlingen, Germany), providing light (LED light source, GDI^®^, São Paulo, Brazil) into the abdominal cavity. A high-definition camera was attached to the laparoscope and connected directly to the HDMI input of a notebook with a 14′′ monitor to view the surgical procedure. Next, the second trocar (10 mm, Ø) was introduced video-assisted into the right inguinal region, approximately 8 cm caudal to the first laparoscopic port and 45° from the medial sagittal plane; the same procedure was performed in the establishment of the third portal, contralateral to the second with a 5 mm Ø trocar. In the second and third ports, atraumatic forceps of 5 and 10 mm Ø, respectively (Babcock^®^, Bhio Supply, Rio Grande do Sul, Brazil), were introduced (Figure 3).

### 2.6. Ovarian Manipulation and Follicular Aspiration

The use of both forceps allowed manipulation of the uterus, bursae, and ovarian tubes and individualization of the ovaries suspended in the mesovarium to avoid rupture of the follicles. 

For follicular aspiration, a vacuum pump (BV-003D^®^, WTA, São Paulo, Brazil) was used with a pressure of 50 mmHg, and a 20 G short-beveled disposable needle (WTA^®^, São Paulo, Brazil) was coupled to the aspiration system. The Teflon tube was 1.7 mm in diameter and 110 cm in length, connected to a 50 mL collection tube sealed with a nontoxic silicone stopper (WTA^®^, São Paulo, Brazil) kept at 38.0 °C.

Before aspiration, the system was washed with a washing solution containing phosphate-buffered saline (PBS) supplemented with 1% fetal bovine serum (FBS) and 10 UI/mL sodium heparin. About 2 mL of this medium was left in the flask that would receive the oocytes. The needle insertion was directed through the abdominal wall using a video-assisted technique to avoid excessive pulling of the ovary. Each ovary was then brought close to the needle for aspiration of visible antral follicular structures regardless of follicular size: small (<6 mm), medium (6–10 mm), or large (>10 mm). After the follicles were perforated, the needle was rotated to ensure the effectiveness of oocyte capture, kept in a position parallel to the ovarian surface, and counted as they were aspirated. In the suction interval between one ovary and the ovaries, the suction system was entirely cleaned by aspiration of the washing solution.

### 2.7. Post-LOPU Lavage, Abdominal Deflation, and Closing

At the end of the procedure, the ovaries were washed with 20 mL of 0.9 NaCl solution containing 2 mL of 2% lidocaine to remove clots formed on the ovarian surface and minimize the formation of adhesions.

Subsequently, CO_2_ was released from the abdominal cavity through the opening of the trocar safety valves, and light manual pressure was applied to the abdominal wall to release gas. The trocars were removed through a rotational movement, allowing the muscle fibers to be reapproximated. Suturing was performed with a Wolff-type dermal suture using Nylon 2-0 (Shalon^®^, Rio de Janeiro, Brazil) without requiring muscle suturing or reducing subcutaneous tissue.

### 2.8. Post-Surgical Management and Evaluation

After litter removal, the calves were placed in a reserved space where they stayed for a short period until they resumed the quadrupedal position. Oxytetracycline 20 mg/kg (Terramycin LA^®^—Zoetis, São Paulo, Brazil) was administered IM in a single dose, and the surgical wounds received a silver sulfadiazine spray solution (Bactrovet^®^ Silver AM, König, São Paulo, Brazil). In the first 6 h after surgery, the pain response behavior of these females was evaluated [14].

### 2.9. Tracking, Counting, and Classification of Cumulus-Oocyte Complexes (COCs)

The tube with aspirated content was sent to a reserved room attached to the surgery site, which served as a field laboratory where the tracking and classification of COCs took place. The aspirated content was deposited on a nylon mesh filter with pores of 75 µm Ø, washed twice, and then deposited in a 60 mm petri dish. The COCs were selected and classified into grades I and II using a stereomicroscope (Nikon^®^, Tokyo, Japan) according to previously established criteria [15].

### 2.10. Statistical Analysis

The duration of each surgical step (AA, PE, OM, and DS), heart and respiratory rates, and rates of oocyte recovery and viable oocytes were analyzed using descriptive statistics (mean ± SD). Statistical analysis was performed using GraphPad Prism 9^®^ software for Windows (Dotmatics, San Diego, CA, USA).

## 3. Results

### 3.1. Fasting and Anesthetic Procedure

**Figure 4:**
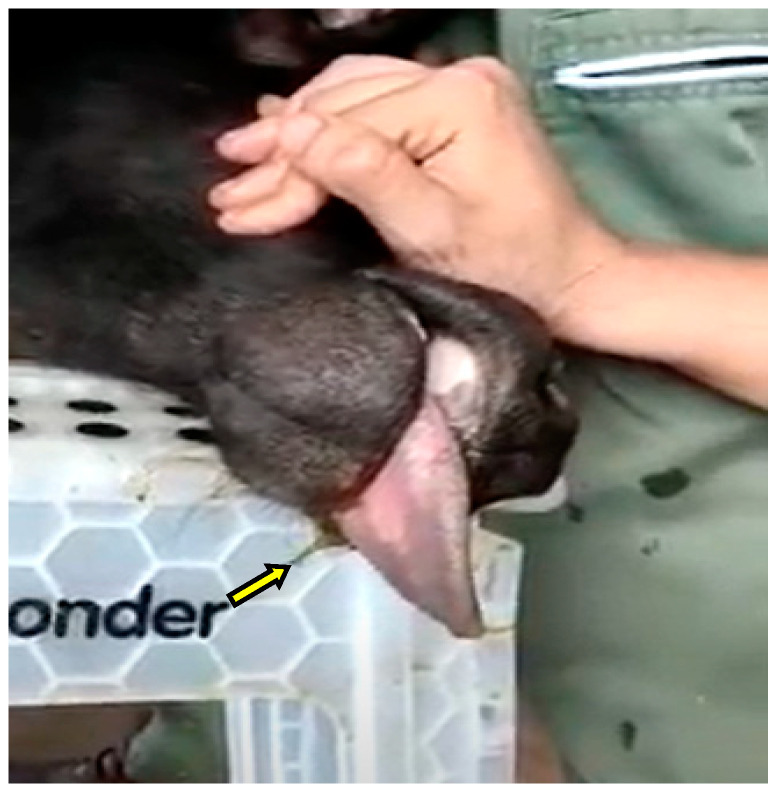
Mild gastroesophageal reflux in a buffalo heifer during the LOPU procedure.

Fasting for 36 h on solids and 24 h on liquids worked efficiently, with gastroesophageal reflux being detected quite discreetly in two LOPUs (11%; 18 LOPUs in total) (Figure 4). 

The anesthetic protocol using sedatives and local anesthesia proved safe and efficient, allowing the LOPU procedure to be performed in the field without any complications. Variations in HR and RR were observed in the first 10 min of sedation and remained low throughout the procedure. The averages recorded were 40.69 ± 8.3 beats/min and 10.17 ± 4.1 breaths/min for HR and RR, respectively. The calves showed good anesthetic recovery, always getting up without difficulty after the procedures and without revealing any discomfort.

### 3.2. Videolaparoscopic Follicular Aspiration

**Figure 5:**
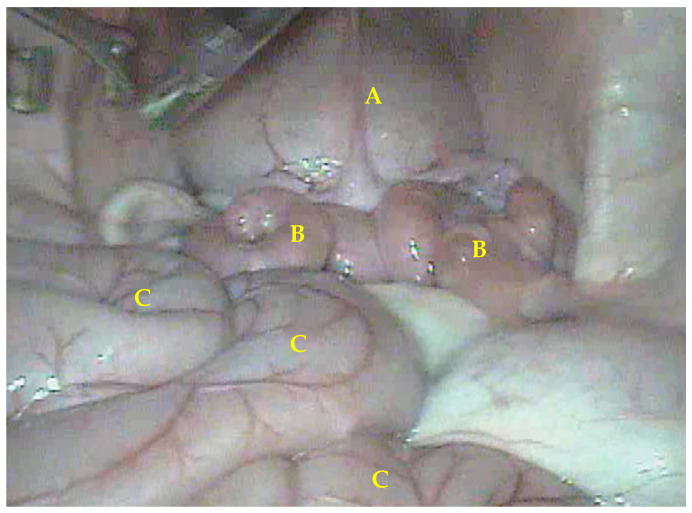
Videolaparoscopic image of the inguinal region of a buffalo heifer undergoing LOPU. Topographic location (craniocaudal view) of uterus (B), cranial to bladder (A); intestines appear in foreground (C).

The intra-abdominal pressure ranged from 5 to 8 mmHg with an insufflation speed of 5 L/min; together with the positioning of the laparoscopic ports, the procedure was efficient and provided excellent visualization of the abdominal cavity. The locations of the uterus and ovaries were determined using the bladder as a reference (Figure 5). In addition, the pneumoperitoneum added to the positioning of the ports allowed an ideal visualization and manipulation of organs in the abdominal cavity.

**Figure 6:**
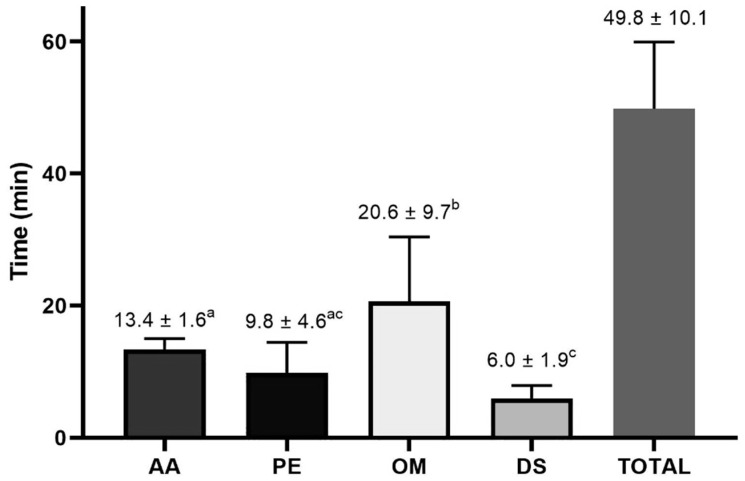
Distribution of time spent in the steps of the LOPU procedure. Anesthetic and antisepsis procedure (AA); establishment of laparoscopic ports (PE); ovarian manipulation and aspiration (OM); washing, deflation, and suturing (DS) Different letters indicate differences between observations (*p* < 0.05).

After 18 procedures, the mean time duration was 49.8 ± 10.1 min. From sedation to the beginning of the surgical procedure (AA), the average time of the procedure was 13.45 ± 1.63 min: establishment of ports and abdominal insufflation (PE), 9.8 ± 4.6 min; ovarian manipulation and aspiration (OM), 20.6 ± 9.7 min; Lavage, deflation, and suturing (DS), 6.0 ± 1.9 min. Significant differences were observed between the times of the established surgical steps (*p* < 0.05), and the manipulation and aspiration of the ovaries (OM) step required the most time for the procedure (Figure 6).

**Figure 7:**
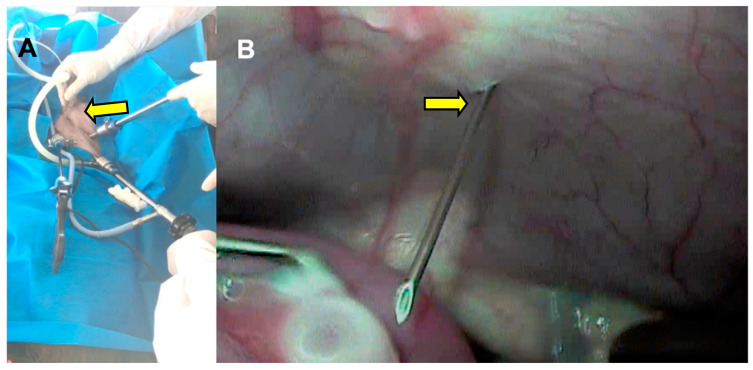
(**A**) Transabdominal use of the aspiration needle (arrow), caudal to the transverse plane formed by the right and left ports. (**B**) Video-assisted entry of the aspiration needle (arrow) into the abdominal cavity.

**Figure 8:**
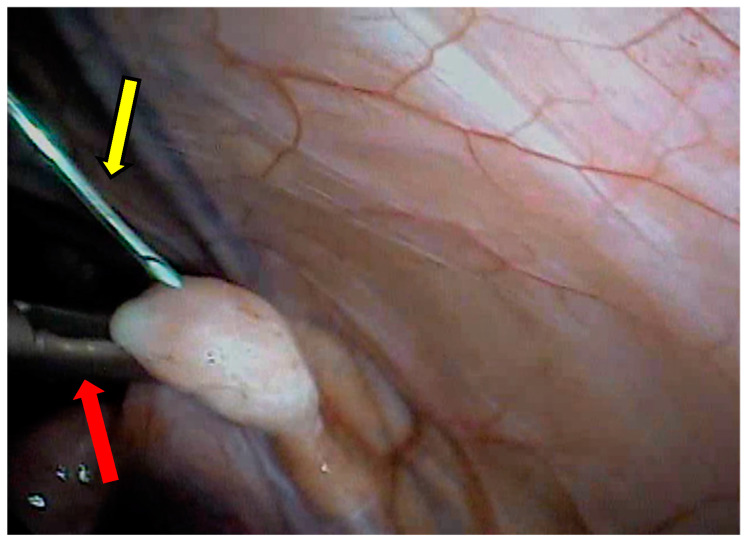
Laparoscopic view of the ovary of a buffalo calf submitted to follicular aspiration. Red arrow, atraumatic forceps; yellow arrow, aspiration needle.

The transabdominal 20 G needle (Figure 7) associated with a pressure of 50 mmHg had a recovery rate of 60.3% (Figure 8).

Discrete ovarian bleeding caused by follicular puncture was also recorded. However, this was controlled before the end of the LOPU procedure and before the final washing of the ovaries for clot removal, preventing adhesion formation. During repeated procedures, no ovarian or uterine alterations were observed in the animals.

**Figure 9:**
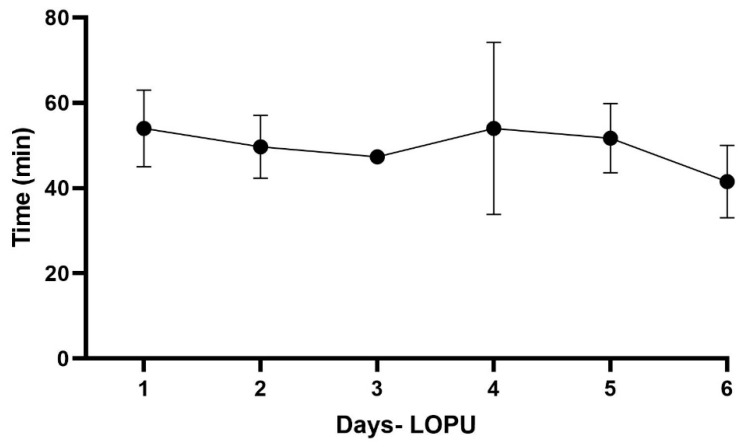
Graphic demonstration of the learning curve of laparoscopic follicular aspiration in buffalo calves. Each point represents the average time involving three procedures/day.

The learning curve was assessed by analyzing the operative time over the days in which the procedures were performed. As shown in Figure 9, despite the small reduction, there was no significant variation in operative time over 6 days after the procedure (*p* > 0.05).

The greatest challenge during the learning process was the adequate grasping of the ovaries so that they would not injure the fallopian tubes and correct follicular puncture without passing through the ovary, thus causing extravasation of its content and increasing the chances of losing the oocyte in the abdominal cavity.

**Figure 10:**
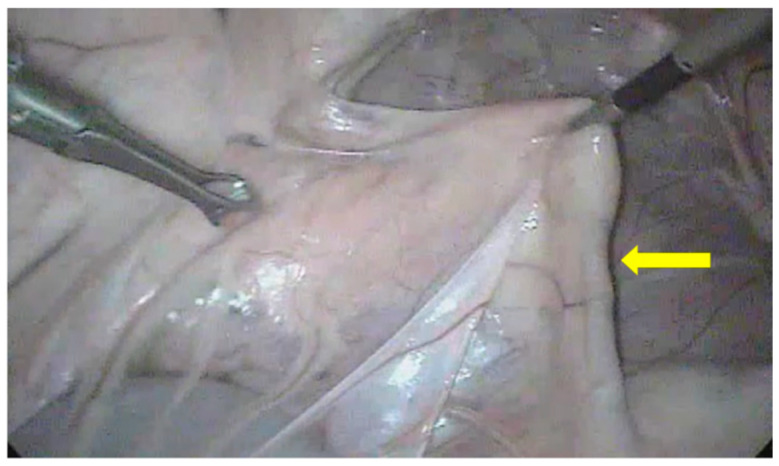
Omentum (arrow) covering the abdominal organs, making the procedure impossible to perform.

One complication that made the procedure difficult and increased surgical time was the omentum covering abdominal organs post insufflation (Figure 10). It was observed that, in four cases (22.2%), during the introduction of the first trocar, this intercurrence delayed the total surgery time by an average of 9 ± 1 min because, after its observation, maneuvers had to be performed to remove the gas and the first trocar, which was then replaced.

### 3.3. Post-Surgical Evaluation

**Figure 11:**
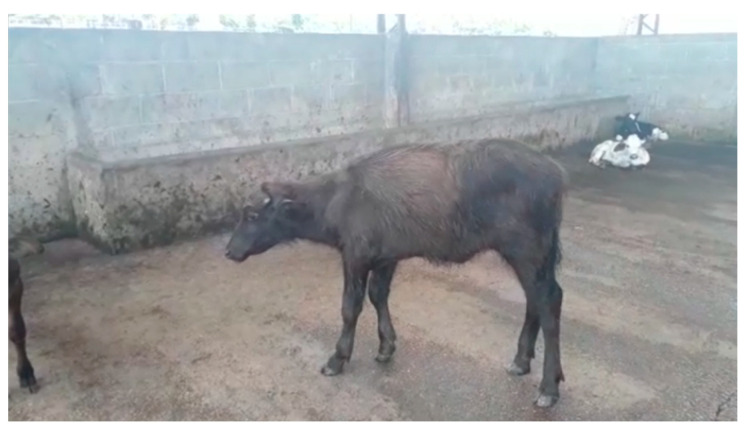
Animal behavior immediately after surgery (time 0). Animal under observation until ready for re-establishment.

A slight sign of ataxia and mild discomfort was observed in all animals in the first 6 h after surgery (Figure 11). None of the animals presented post-surgical complications, infections, or adhesions that compromised their life and reproduction.

### 3.4. Tracking and Classification of Cumulus–Oocyte Complexes (COCs)

After 18 procedures, 126 follicles (7.17 ± 7.17 follicles aspirated/procedure) of these 76 oocytes were retrieved (4.2 ± 7.1 oocytes retrieved/procedure), visualized, and aspirated. Of the oocytes obtained, 31 (40.8%) were grades I and II, with a mean of 1.72 ± 4.2 oocytes/procedure, not generating transferable embryos. The final results of the LOPU per calf and its individual variations are shown in Table 1.

## 4. Discussion

Animal fasting for 36 and 24 h for solids and liquids, respectively, contributed to the prevention of gastroesophageal reflux [16], as previously reported in goats [17] and sheep [18]. Moreover, it facilitated the observation and manipulation of the organs in the abdominal cavity since there was no interference with the abomasal and vesical volume [16]. In previous procedures performed among buffalo heifers, 24 h fasting for grass and only 12 h fasting for water obtained favorable results [11,19]. However, this was not replicated in this trial. In a pilot study, gastroesophageal reflux was observed in an animal that underwent 24 h fasting. After this occurrence, the fasting of solids was changed to 36 h, maintaining the water restriction for 24 h to preserve the health of the animal.

The good results obtained with xylazine use in keeping the cardiac and respiratory parameters of the animals within the expected range [13,16], as well as good post-surgical recovery, can considerably improve the applicability of the LOPU procedure in buffalo calves, becoming an option in extensive conditions; however, the use of an inhalant anesthesia machine is highly recommended when possible [11,12,20]. Furthermore, lidocaine hydrochloride application was mandatory in this protocol, as xylazine alone did not provide the complete analgesia necessary for performing the LOPU technique [21,22].

For the first portal establishment, despite using the Trendelenburg position to make the procedure safer by maintaining the organs more cranially through gravity [23], it was still necessary to introduce the trocar at a low angle to avoid hitting any organs, as performed in sheep and goats [6,17]. Despite its thinness, the abdominal skin presents great resistance to the first trocar; thus, it must be ensured that it is under the omentum before starting the insufflation. Although this does not directly cause harm to the animal, it is undesirable due to the increase in surgical time. To avoid this, the laparoscope can be used to confirm that the trocar is under the omentum before starting abdominal insufflation. Therefore, this can be considered a low-risk intercurrence, which, in this experiment, influenced only the procedure time [24,25].

The intra-abdominal pressure and inflation rate were considered adequate for establishing pneumoperitoneum, and there was no animal discomfort or respiratory difficulty, in addition to allowing visualization of the uterus and ovaries of the calves, similar to those observed by Teixeira [25].

As a variation to the technique which uses two grasping forceps according to Teixeira [7], we believe that this approach makes the manipulation and ovarian positioning procedure faster and easier, especially for less experienced professionals. However, during aspiration, with the ovary properly seized, only one grasping forceps is necessary.

The total surgical time was similar to that obtained in sheep and goat species at 35 min [17] and 26.75 ± 9.6 min [6], respectively, since this study included the total duration of the anesthetic and antisepsis procedures. The extended period spent in the manipulation and follicular aspiration (MO) stage showed that this was the most complex process of the LOPU, and it could vary according to the follicle number presented, as demonstrated in previous studies [25,26]. The learning curve showed that 18 procedures were enough to have the necessary expertise for professional individuals responsible for the LOPU, similar to that obtained in renal biopsies guided by laparoscopy in dogs and swine [27] and even in sheep LOPU [6].

The suction set consisting of a 20 G needle and an aspiration pressure of 50 mmHg is similar to that used in buffalo species, in both adult and prepubertal individuals [11,12,28]. The recovery rate was below the 84.3% ± 29.3% obtained in buffalo calves [12] but within the results obtained in small ruminants [17,25]. However, the quality of oocytes recovered was lower than that observed in Mediterranean buffalo heifers with 16.2 ± 9 viable oocytes [11].

## 5. Conclusions

In summary, LOPU was proven to be an effective technique for oocyte retrieval among buffalo heifers, with a good oocyte recovery rate and a short execution time. In addition, the sedation procedure combined with local anesthesia was efficient, without intercurrences, making it possible to perform the technique in farm conditions, thus eliminating the need for a sterile hospital environment and the use of an inhalant anesthesia machine, and reducing the cost of the procedure without causing risks to the health of the animals as long as the necessary care regarding asepsis is observed. Altogether, IVEP with a practical LOPU in buffalo heifers is expected to be part of the reproductive biotechnology toolbox for buffalo farmers, with particular advantages of increasing reproduction in prepubertal animals, decreasing the interval between generations, and accelerating the genetic gain in the species.

## Figures and Tables

**Table 1:** Summary of procedures performed on six buffalo heifers with a 15 day interval between LOPUs.

	Aspirated Follicles				Retrieved Oocytes				Viable Oocytes			
Animal/LOPU	1st	2nd	3rd	Average	1st	2nd	3rd	Average	1st	2nd	3rd	Average
1	9	4	4	5.7 ± 2.4	4	2	1	2.3 ± 1.2	3	1	0	1.3 ± 1.2
2	7	9	5	7.0 ± 1.6	7	2	3	4.0 ± 2.2	2	1	1	1.3 ± 0.5
3	6	4	5	5.0 ± 0.8	3	2	2	2.3 ± 0.5	2	0	1	1.0 ± 0.8
4	5	3	3	3.7 ± 0.9	2	1	3	2.0 ± 0.8	1	0	0	0.3 ± 0.5
5	4	2	0	2.0 ± 1.6	2	0	0	0.7 ± 0.9	1	0	0	0.3 ±0.5
6	33	13	10	18.7 ± 10.2	31	1	10	14.0 ± 2.6	18	0	0	6.0 ± 8.5
Total	64	35	27	7.0 ± 2.9	49	8	19	4.2 ± 3.0	27	2	2	1.7 ± 2.0
